# 

**Published:** 1909-12

**Authors:** 


### BOOKS RECEIVED.

Clinical Studies for Nurses: For Second and Third Year Pupil Nurses. By Charlotte A. Aikens, formerly Superintendent of Columbia Hospital, Pittsbury, and of Iowa Methodist Hospital, Des Moines. 12 mo of 510 pages, illustrated. Philadelphia and London: W. B. Saunders Company. 1909. (Cloth, $2.00 net.)

Surgical Diagnosis. By Daniel N. Eisendrath, M.D., Professor of Surgery in the Medical Department of the University of Illinois (College of Physicians and Surgeons). Second revised edition. Octavo of 885 pages, with 574 original illustrations, 25 in colors. Philadelphia and London: W. B. Saunders Company. 1909. (Cloth, $6.50; half morocco, $8.00, net prices.)

Clinical Examination of the Urine and Urinary Diagnosis. By J. Bergen Ogden, M.D., Medical Chemist to the Metropolitan Life Insurance Company, New York. Third edition, revised. Octavo of 427 pages, illustrated. Philadelphia and London: W. B. Saunders Company. 1909. (Cloth, $3.00 net.)

A Treatise on Diseases of the Nose, Throat and Ear. By William Lincoln Ballenger, M.D., Professor of Laryngology, Rhinology and Otology in the College of Physicians and Surgeons, Chicago. New (2) edition, thoroughly revised. Octavo, 930 pages, with 491 engravings, mostly original, and 17 colored plates. Lea & Febiger, Philadelphia and New York. 1909. (Cloth, $5.50, net.)

The Medical Complications, Accidents and Sequels of Typhoid Fever and the Other Exanthemata. By H. A. Hare, M.D., B.Sc., Professor of Therapeutics in the Jefferson Medical College and Physician to the Jefferson College Hospital, Philadelphia, and E. J. G. Beardsley, M.D., L.R.C.P., Philadelphia. With a special chapter on the Mental Disturbances Following Typhoid Fever, by F. X. Dercum, M.D., Professor of Nervous Diseases in the Jefferson Medical

College. Second edition, thoroughly revised and much enlarged. Octavo, 398 pages, with 26 engravings and 2 plates. Lea & Febiger, Philadelphia and New York, 1909. (Cloth, $3.25, net.)

The Practical Medicine Series. Ten volumes. Under the general editorial charge of Gustavus P. Head, M.D., Professor of Laryngology and Rhinology in the Chicago Post-Graduate Medical School. Vol. VIII. Materia Medica and Therapeutics, Preventive Medicine, and Climatology, edited by George F. Butler, M.D., Henry B. Flavill, M.D., and Norman Bridge, M.D. Series 1909. Chicago: The Year Book Publishers. (Price, $1.50; entire series, $10.00.)

Diseases of Infants and Children. By Henry Dwight Chapin, A.M., M.D., Professor of Diseases of Children, New York Post-graduate Medical School and Hospital. And Godfrey Roger Pisek, M.D., Professor of Diseases of Children, University of Vermont. Octavo, pp. 624. With 179 illustrations and 11 colored plates. New York: William Wood & Co. (Cloth, $4.50 net.)

Diseases of Children. By Henry Enos Tuley, M.D., Professor of Obstetrics, University of Louisville. Octavo, pp. 662. Illustrated. Baltimore: Southern Publishing Co. 1909. (Cloth, $5.00; half leather, $6.00.)

Transactions of the American Urological Association. Seventh annual meeting held at Chicago, June 1 and 2, 1908. Edited by Charles Greene Cumston, M.D.

Transactions of the American Otological Society. Forty-second annual meeting held at Harvard Medical School, Boston, June 1 and 2, 1909. Vol. XL. Part II. J. F. McKernon, M.D., Secretary.

Transactions of the American Dermatological Association. Thirty-second annual meeting held at Annapolis, Md., September 24 and 25, and at Baltimore, September 26, 1908. Grover W. Wende, M.D., Secretary.

Medical Directory of New York, New Jersey and Connecticut, published by the Medical Society of the State of New York. 1909.

